# Osteogenesis imperfecta type III: Oral, craniofacial characteristics and atypical radiographic findings oral

**DOI:** 10.4317/jced.58263

**Published:** 2021-10-01

**Authors:** Camila Arantes, Isabela Sica, Milena Bezerra, Cristhiane Amaral, Caio Bellato, Gustavo Logar

**Affiliations:** 1 Graduate Program in Dentistry, University of Western São Paulo, Presidente Prudente, Brazil; 2 DDS, Ms, Department of Special Care Dentistry, Dental School, University of Western Sao Paulo, Presidente Prudente, Sao Paulo, Brazil; 3 DDS, MS, Department of Surgery, University of Western Sao Paulo, Presidente Prudente, Sao Paulo, Brazil

## Abstract

Osteogenesis imperfecta (OI) is a disease characterized by decreased bone mineral density, causing susceptibility to bone fractures by mild trauma and bone deformities. The aim of this study was to describe an osteogenesis imperfecta type III clinical case, its craniofacial and oral changes as well as its atypical radiographic findings. An eighteen-year-old, male patient diagnosed with osteogenesis imperfecta type III was referred for dental evaluation; the clinical examination showed the craniofacial and oral changes of the disease such as triangular face, class III malocclusion, anterior open bite and posterior crossbite, dentinogenesis imperfecta presenting amber discoloration. The radiographic examination revealed teeth with pulp chamber obliteration and root canals, however unusual findings were also observed such as: bilateral increase of the mandibular canals and preservation of the pulp chamber and third molar root canals. Our findings show that is essential an adequate knowledge of anatomy, a careful anamnestic evaluation and a complete radiological evaluation of the patient with OI.

** Key words:**Dental anomalies, developmental disability, rare disorders.

## Introduction

Osteogenesis imperfecta (OI) is a disease caused by autosomal dominant genetic alterations of connective tissue which affect the structure and function of type I collagen (1). This disease is characterized by decreased bone mineral density, causing susceptibility to bone fractures by mild trauma and bone deformities. Moreover, in some cases it is observed short stature, hearing impairment, blue sclera, ligament laxity, joint hypermobility and dentinogenesis imperfecta as they affect tissues rich in collagen such as tendons, cornea, dentin, sclera, fascia, among others (2-4).

Regarding craniofacial alterations it presents increased encephalic diameter, triangular facial appearance and mandibular protrusion and retrusion of the middle third of the face, with high incidence of class III malocclusion, anterior and posterior cross bite and open bite (3).

It is a rare disease that occurs from 1 to 10,000 live births, with a prevalence of 1 in 200,000 individuals (1,4). The treatment is performed through bisphosphonates which interfere in osteoclastic activity as following: prevention of osteoclast formation, inhibition of osteoclastic action and early apoptosis of osteoclasts. This decreased osteoclastic activity reduces pain, increases cortical bone thickness and bone density, as well (5).

Therefore, the purpose of this study is to describe the craniofacial and oral manifestations of an osteogenesis imperfecta type III patient as well as atypical radiographic findings not reported in the literature up to the present moment.

## Case Report

An eighteen-year-old male, wheelchair user, referred to Universidade do Oeste Paulista Dentistry Faculty clinic. The patient reported being diagnosed with osteogenesis imperfecta type III after episodes of bone fractures during childhood and adolescence. The treatment chosen was Pamidronate 1000 UN / 1L in serum for four hours every three months for 4 years, from 11 to 15 years of age. Since then the patient has not presented other bone fractures.

The general clinical characteristics of the osteogenesis imperfecta observed in this patient were: short stature (1.34m), deficiency in the growth of the lower limbs and kyphoscoliosis, however, neither hearing nor the pulmonary and cardiovascular system were affected. The extra-oral clinical examination showed the patient’s triangular face and a conjunctival sclera with slight bluish color.

**Figure 1:**
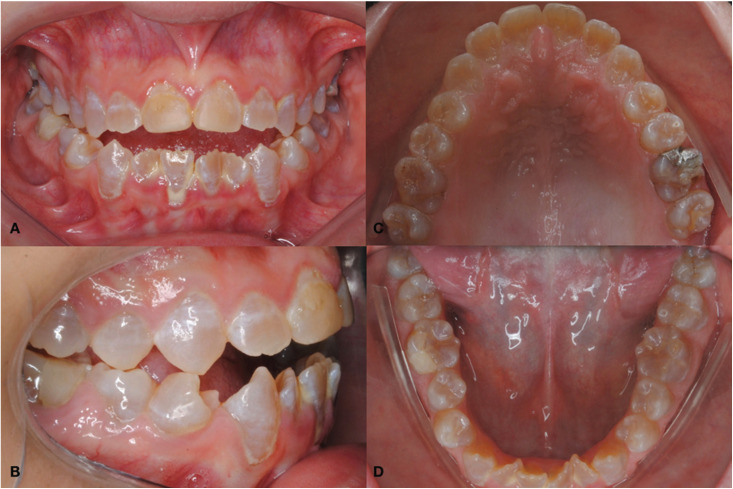
Intra oral clinical examination. A: Anterior open bite and brownish teeth. B: Angle Class III and posterior crossbite. C: tooth 26 with amalgam restoration crack. D: anterior teeth presenting crowding and dental calculus.

The intra oral clinical examination showed class III angle occlusion, anterior open bite, posterior cross bite anterior teeth with crowding, dentinogenesis imperfecta presenting amber discoloration, crack in mesial occlusal amalgam restoration on tooth 26, presence of gingivitis and dental calculus (Fig. 1).

**Figure 2:**
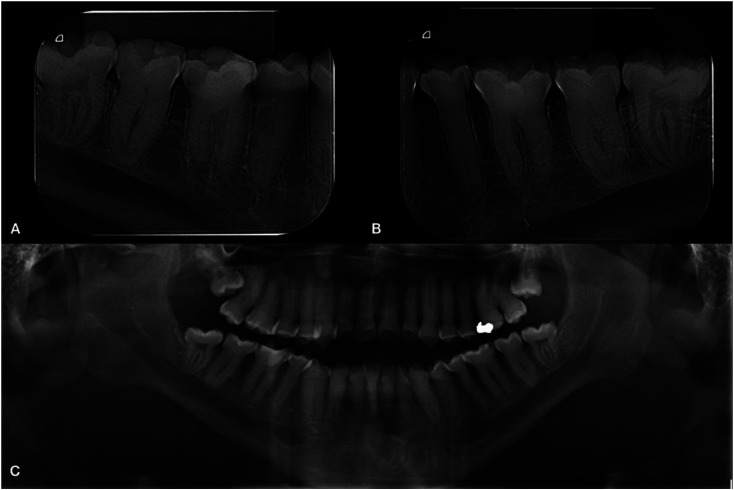
Radiographic examination. A, B: Periapical radiographic showing the teeth with absent pulp chamber and obliterate root canals (third molar exception) and bilateral mandibular canal increase, right and left side. C: Panoramic radiographic showing these findings.

**Figure 3:**
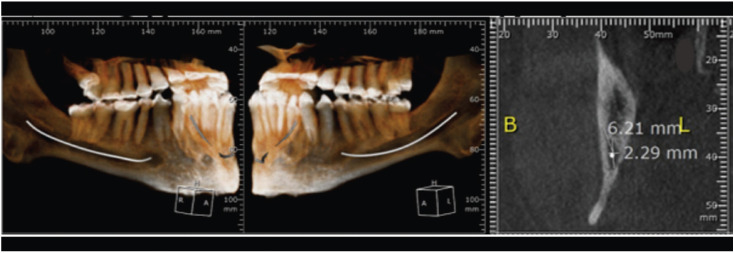
Tomographic examination. A: Mandibular canal. B: Increase in the mandibular canal height in the region near the lingula.

However, the radiographic examination showed teeth with absence of pulp chamber presenting root canals obliteration, except for the third molars. In addition, a bilateral increase of the mandibular canal was found (Fig. 2). This increase in the mandibular canal was confirmed by a cone beam CT scan with 1 mm transversal cuts showing a mandibular canal with a height variation from 6.21 mm to 3.9 mm in its extension (Fig. 3).

Based on anamnesis and clinical examination, a treatment plan was drawn up. At first, oral prophylaxis was performed to remove the dental calculus, followed by oral hygiene instructions. After improvement of the initial condition, in the subsequent consultation, the amalgam restoration was exchanged on tooth 26, which had a crack.

## Discussion

The more severe the osteogenesis imperfect, more severe the Angle class III malocclusions, anterior open bite, crossbite posterior and mandibular prognathism that may be related to a lack of antero-inferior displacement of the maxilla caused by a primary growth defect at the base of the skull combined with a vertical underdevelopment of the alveolar structures and the condylar process (3).

Regarding the dental anomalies, besides dentinogenesis imperfecta, the dental agenesis is reported in 17% of individuals with osteogenesis, 43% of which are related to OI type III. This is because the abnormal type I collagen of these patients has a deleterious effect on the dental germ development in this region during the onset of mineralization (4). The patient in the report presented all permanent teeth, confirmed by the clinical and radiographic examination, being an exam simple, efficient and low cost.

An atypical radiographic alteration observed in the patient was the bilateral increase of mandibular canal height, which varied from 6.21 mm in the region of the lingula to 3.9 mm close to the foramen mentonian. It is the first finding in the literature in patients with osteogenesis imperfecta. Other studies have reported this increase in patients presenting malignant neoplasias and osteomyelitis and in all these cases the canal 12-16 increase is unilateral. According to Ai *et al*., 2017 (6), these alterations can be observed in patients with benign neoplasms, arteriovenous malformations, radiographic artifacts and anatomical variations, and in their study of clinical cases there has been a variation of 5.1mm to 8.8mm of unilateral increase of the mandibular canal, the normal canal size is 2 to 2.4 mm.

Concerning OI patients this clinical case demonstrated to be unique and it occurs bilaterally therefore it is difficult to define whether it is a disease change, an arteriovenous malformation or yet a change after bisphosphonate use.

The main dental change in the radiographic examination of OI patients is dentinogenesis imperfecta which shows partial or total obliteration of the pulp chamber and root canals, short and thin roots (1). Except for the short and thin roots, the other aspects were observed in the case of our study. However, a radiographic finding of the third molars is worth mentioning once it is the only report in OI patients who present dentingenesis imperfecta showing the radiographic pulp chamber image and preserved root canals without obliteration.

The third molar formation can occur between 10 and 15 years old (7), the same age the patient reported using Pamidronate. One hypothesis of the normal formation of these teeth would be the performance of this drug in dentinogenesis. The only study that shows this relationship is by Yamaza *et al*., 2018 (8), in human stem cells culture exfoliated by Pamidronate and it was concluded that this drug can prevent dentinal abnormalities.

OI patients use bisphosphonates as a treatment, and these drugs act directly in order to prevent the osteoclast recruitment by the bone tissue, inhibiting the action of the ones already present in the bone or reducing their half-life by apoptosis (2). They can act indirectly in the osteoblasts that will prevent the bone tissue of recruiting the osteoclasts, resulting in decreased fractures, improved strength and muscle mobility and thus enhance well-being (9).

Despite the benefits of these drugs, one of the important side effects affecting the oral cavity is the jaw osteonecrosis which can occur after an invasive dental procedure or even spontaneously (9).

That said, it is important to emphasize and value preventive treatment in these patients, since their compromised systemic condition may cause even greater damage if subjected to more invasive treatments such as tooth extractions, as well as their local condition that makes endodontic treatment quite complicated for the absence of pulp chamber and obliteration of root canals in dental elements. (10) Another important factor to be highlighted is the quality of the adhesion to the enamel and dentin in these teeth with imperfect dentinogenesis, it is assumed that there is no adequate formation of the hybrid layer and this would compromise the quality and useful life of composite resin restorations (11), therefore the amalgam restoration was replaced by the same material that already existed.

Our findings show that is essential an adequate knowledge of anatomy, a careful anamnestic evaluation and a complete radiological evaluation of the patient with OI. In general, a multidisciplinary approach to the management of this atypical characteristics is more beneficial, with care centered on prevention and maximizing the patients quality of life.
